# Pure primary ovarian squamous cell carcinoma: A case report and review of the literature

**DOI:** 10.3892/ol.2014.2650

**Published:** 2014-10-31

**Authors:** JUNG-WOO PARK, JONG WOON BAE

**Affiliations:** Department of Obstetrics and Gynecology, Dong-A University, College of Medicine, Busan 602812, Republic of Korea

**Keywords:** ovary, ovarian carcinoma, pure primary squamous cell carcinoma, dermoid cyst, endometriosis

## Abstract

Pure primary ovarian squamous cell carcinoma (SCC) is a rare lesion that usually arises from the malignant transformation of an existing ovarian dermoid cyst. The *de novo* occurrence of an ovarian SCC in the absence of a prior ovarian dermoid cyst, Brenner tumor or endometriosis is extremely rare. At present, no effective therapy exists for treating pure primary ovarian SCC. The present case study describes a patient that presented with progressive coughing, who was diagnosed with an International Federation of Gynecology and Obstetrics stage IV pure primary ovarian SCC with lung metastases. The patient received postoperative chemotherapy, however, the patient succumbed to the disease. The current study also presents a review of the literature.

## Introduction

The incidence of a pure primary ovarian squamous cell carcinoma (SCC) is extremely rare when not associated with pre-existing ovarian lesions, such as dermoid cysts, Brenner tumors or endometriosis (whose presence is normally indicative of ovarian SCC) (1). To date, only 30 cases of pure primary ovarian SCC have been reported worldwide. Due to the rarity of pure primary ovarian SCC, the clinical features of the disease have not been established and effective treatments are yet to be identified. Subsequent to optimal tumor devulking, patients with early-stage pure primary ovarian SCC may remain disease-free. However, those patients with advanced-stage disease may experience a poorer outcome, despite treatment with postoperative chemotherapy and/or radiotherapy. The present case study describes a patient with pure primary ovarian SCC, and presents a review of the literature. Written informed consent was obtained from the patient’s family.

## Case report

A 46-year-old female (gravida 3, para 2) was referred to the Department of Pulmonary Medicine (Dong-A University, College of Medicine, Busan, Republic of Korea) with a history of progressive coughing that had been apparent for three months. Upon chest computed tomography (CT), performed at a local clinic on February 11, 2012, a diagnosis of lung cancer was suspected. The patient was immediately hospitalized and underwent bronchoscopy. A transbronchial lung biopsy revealed an SCC of unknown primary site.

To locate the primary site of the metastatic lung cancer, an abdominal CT and positron emission tomography-CT scan was performed. The results of the scans revealed a left-sided pelvic mass, a left hydronephrosis and multiple regions of lymph node metastasis in the pelvic, abdominal, mediastinal and supraclavicular areas.

The patient was referred to the Department of Obstetrics and Gynecology (Dong-A University, College of Medicine) for gynecological treatment. Abdominal exploration was performed on February 24, 2012, which revealed that the solid mass arose from the left adnexal area, was densely adhered to the sigmoid colon and external iliac vessel, and was encapsulating the left ureter. A total abdominal hysterectomy, bilateral salpingo-oophorectomy, adhesiolysis between the tumor and sigmoid colon, segmental resection of the sigmoid colon and reanastomosis were performed. Following segmental resection, the patient underwent an end-to-end anastomosis of the left ureter.

**Figure 1:**
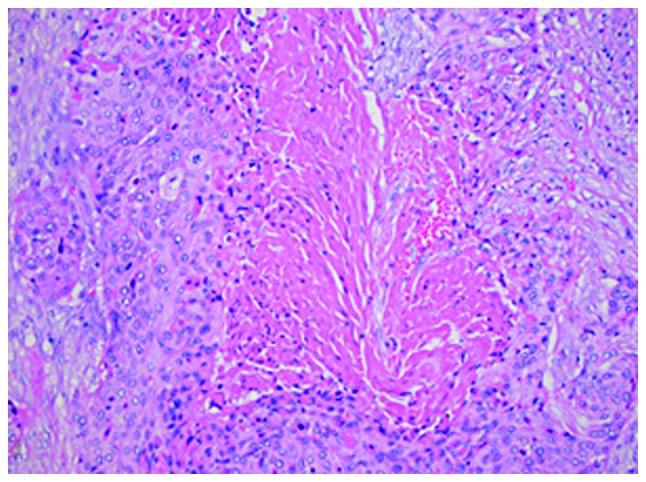
Histopathological staining revealing a pure squamous cell carcinoma arising from the left ovary, a notable observation in the absence of any concomitant dermoid cyst or endometriosis (stain, hematoxylin and eosin; magnification, ×200).

Histopathological analysis confirmed a pure SCC arising from the left ovary. The pathological results were notable for the absence of any associated dermoid cyst or features suggestive of endometriosis, (Fig. 1). The patient was subsequently diagnosed with stage IVB pure primary ovarian SCC with lung metastases according to the International Federation of Gynecology and Obstetrics staging system (2). Following surgery, the patient was administered a six-course adjuvant chemotherapy regimen, consisting of paclitaxel (175 mg/m^2^) and carboplatin (5 mg/ml/min) at three-week intervals. Despite the initiation of first-line adjuvant chemotherapy, clinical and radiographical evidence identified tumor progression and aggravation of the lung metastasis. Therefore, a second-line three-course chemotherapy regimen, consisting of topotecan (1 mg/m^2^) and cisplatin (50 mg/m^2^) at three-week intervals, and a third-line three-course regimen, consisting of etoposide and ifosfamide at three-week intervals, was administered. Despite this, treatment was unsuccessful and the patient succumbed to the disease on February 12, 2013, following cardiopulmonary arrest.

## Discussion

Primary ovarian SCC is rare, with the majority of cases preceded by dermoid cysts. Alternatively, cases of primary ovarian SCC may be associated with Brenner tumors and endometriosis (3). Ovarian SCCs that are reported to arise within a dermoid cyst appear to be incidental histological findings (4). In total, ~2% of cases of primary ovarian SCC originate from the malignant transformation of a dermoid cyst (5). A previous study of metastatic ovarian tumors revealed that a total of 2.5% are of the squamous cell type, with the majority of cases of metastatic SCC originating by direct extension from the cervix (6). Furthermore, of the reported cases of pure ovarian SCC, the most significant association identified was with cervical dysplasia (7,8). However, this association was not identified in the present case study, as revealed by a negative pre-operative pap smear and by post-operative pathological analysis. The incidence of pure primary ovarian SCC is extremely low, with thirty cases described by previous studies (Table I) (1,3,7–21). Previous studies revealed that stage and grade of tumors correlate with overall survival in pure primary ovarian SCC patients. Thus, patients with early-stage pure primary ovarian SCC may remain disease-free after optimal debulking. However, those patients with advanced-stage disease may experience a poorer outcome, despite treatment with post-operative chemotherapy and/or radiotherapy (7). Due to the rare nature of pure primary ovarian SCC, effective adjuvant chemotherapy or radiotherapy regimens have not yet been established. In the present case study, the patient was unresponsive to the chemotherapy regimen administered following surgical debulking. In the twelfth month subsequent to surgery, the patient succumbed to the rapidly-progressive disease. The chemotherapy regimens administered in the present study, or the doses used, may be unsuitable for this ovarian malignant cell type. Therefore, to identify effective therapies for the treatment of pure primary ovarian SCC, further clinical investigations are required.

## Figures and Tables

**Table I:** Clinicopathological features of pure primary squamous cell carcinoma reported in the literature.

First author/s (ref.)	Year	Case	Age, years	FIGO stage	Grade	Treatment	Follow-up, months
Genadry *et al* (11)	1979	1	41	CIS	1	TAH, BSO	NR
McGrady *et al* (12)	1993	2	53	CIS	1	TAH, BSO	Alive
Sworn *et al* (13)	1995	3	39	CIS	3	TAH, BSO	Alive, 60
Yetman and Dudzinski (8)	1989	4	33	I	2	TAH, BSO	Alive, 15.6
Black and Benitez (14)	1964	5	35	I	1	TAH, BSO	NR
Shingleton *et al* (15)	1974	6	54	I	1	RO, RT	DOD, 6
Mai *et al* (16)	1996	7	40	I	2	TAH, BSO	NR
Macko and Johnson (17)	1983	8	90	I	2	UO	Alive, 30
Chen (18)	1988	9	49	I	1	TAH, BSO, RT	Alive, 12
Balat *et al* (19)	2001	10	40	IB	NR	TAH, BSO, PLND, appendectomy, right nephrectomy, chemotherapy	DOD, 24
Kashimura *et al* (9)	1989	11	61	II	NR	TAH, BSO, RT, chemotherapy	DOD, 9
		12	42	III	NR	LSO, RT	DOD, 8
		13	50	I	NR	TAH, BSO, RT	Alive, 14.4
Pins *et al* (7)	1996	14	73	IIA	3	TAH, BSO, RT	DOD, 49
		15	61	IIB	3	TAH, BSO, RT, chemotherapy	Alive, 60
		16	55	IIB	3	TAH, BSO, TD, chemotherapy	Alive, 30
		17	38	IIC	3	TAH, BSO, chemotherapy	DOD, 8
		18	64	IB	2	RSO, LO	AWD, 60
		19	55	IIIB	3	TAH, BSO, chemotherapy	DOD, 2
		20	52	IIIC	3	Ovarian, omental biopsy	NR
		21	46	IIIC	3	Ovarian, omental biopsy	NR
		22	27	IIIC	3	TAH, BSO, chemotherapy	DOD, 1
		23	70	IIIC	3	TAH, BSO, chemotherapy	DOD, 5
		24	73	IV	3	LSO, RT	DOD, 1
Ben-Baruch *et al* (3)	1988	25	65	III	2	TAH, BSO, iliectomy, TD, chemotherapy	DOD, 6
Amjad and Pal (20)	2008	26	31	IIIC	1	TAH, BSO, TO, bowel resection, chemotherapy	AWD, 1
Radhi and Awad (10)	1990	27	64	IV	2	TD	DOD, 9 days
Chien *et al* (21)	2005	28	63	IV	3	TAH, BSO, PLND, TO, TD	DOD, 7
Park *et al* (1)	2010	29	76	IIC	1	TAH, BSO, PLND, PALND, TO, appendectomy, chemotherapy	Alive, 42
		30	48	IV	2	TAH, BSO, PLND, PALND, TO, appendectomy, chemotherapy	Alive, 6
Present case	2014	31	46	IVB	2	TAH, BSO, TD, bowel resection	DOD, 12

FIGO, International Federation of Gynecology and Obstetrics; TAH, total abdominal hysterectomy; BSO, bilateral salpingo-oophorectomy; RO, right oophorectomy; RT, radiation therapy; UO, unilateral oophorectomy; PLND, pelvic lymph node dissection; LSO, left salpingo-oophorectomy; TD, tumor debulking; RSO, right salpingo-oophorectomy; LO, left oophorectomy; TO, total omentectomy; PALND, para-aortic lymph node dissection; NR, not recorded; DOD, died of disease; AWD, alive with disease; CIS, carcinoma *in situ*.

## References

[1] Park JY, Song JS, Choi G, Kim JH, Nam JH (2010). Pure primary squamous cell carcinoma of the ovary: a report of two cases and review of the literature. Int J Gynecol Pathol.

[2] Mutch DG, Prat J (2014). 2014 FIGO staging for ovarian, fallopian tube and peritoneal cancer. Gynecol Oncol.

[3] Ben-Baruch G, Menashe Y, Herczeg E, Menczer J (1988). Pure primary ovarian squamous cell carcinoma. Gynecol Oncol.

[4] Dos Santos L, Mok E, Iasonos A (2007). Squamous cell carcinoma arising in mature cystic teratoma of the ovary: a case series and review of the literature. Gynecol Oncol.

[5] Peterson WF (1957). Malignant degeneration of benign cystic teratomas of the ovary; a collective review of the literature. Obstet Gynecol Surv.

[6] Webb MJ, Decker DG, Mussey E (1975). Cancer metastatic to the ovary: factors influencing survival. Obstet Gynecol.

[7] Pins MR, Young RH, Daly WJ, Scully RE (1996). Primary squamous cell carcinoma of the ovary. Report of 37 cases. Am J Surg Pathol.

[8] Yetman TJ, Dudzinski MR (1989). Primary squamous carcinoma of the ovary: a case report and review of the literature. Gynecol Oncol.

[9] Kashimura M, Shinohara M, Hirakawa T, Kamura T, Matsukuma K (1989). Clinicopathologic study of squamous cell carcinoma of the ovary. Gynecol Oncol.

[10] Radhi JM, Awad SM (1990). Bilateral squamous cell carcinoma of the ovary. Case report. Br J Obstet Gynaecol.

[11] Genadry R, Parmley T, Woodruff JD (1979). Secondary malignancies in benign cystic teratomas. Gynecol Oncol.

[12] McGrady BJ, Sloan JM, Lamki H, Fox H (1993). Bilateral ovarian cysts with squamous intraepithelial neoplasia. Int J Gynecol Pathol.

[13] Sworn MJ, Jones H, Letchworth AT, Herrington CS, McGee JO (1995). Squamous intraepithelial neoplasia in an ovarian cyst, cervical intraepithelial neoplasia, and human papillomavirus. Hum Pathol.

[14] Black WC, Benitez RE (1964). Nonteratomatous squamous-cell carcinoma in situ of the ovary. Obstet Gynecol.

[15] Shingleton HM, Middleton FF, Gore H (1974). Squamous cell carcinoma in the ovary. Am J Obstet Gynecol.

[16] Mai KT, Yazdi HM, Bertrand MA, LeSaux N, Cathcart LL (1996). Bilateral primary ovarian squamous cell carcinoma associated with human papilloma virus infection and vulvar and cervical intraepithelial neoplasia. A case report with review of the literature. Am J Surg Pathol.

[17] Macko MB, Johnson LA (1983). Primary squamous ovarian carcinoma. A case report and review of the literature. Cancer.

[18] Chen KT (1988). Squamous cell carcinoma of the ovary. Arch Pathol Lab Med.

[19] Balat O, Aydin A, Camci C, Kutlar I, Büyükberber S (2001). Bilateral primary squamous cell carcinoma of the ovary: a case report of isolated metastasis to the lateral pelvic wall. Eur J Gynaecol Oncol.

[20] Amjad AI, Pal I (2008). De novo primary squamous cell carcinoma of the ovary: a case of a rare malignancy with an aggressive clinical course. J Pak Med Assoc.

[21] Chien SC, Sheu BC, Chang WC, Wu MZ, Huang SC (2005). Pure primary squamous cell carcinoma of the ovary: a case report and review of the literature. Acta Obstet Gynecol Scand.

